# RNA secondary structure prediction using an ensemble of two-dimensional deep neural networks and transfer learning

**DOI:** 10.1038/s41467-019-13395-9

**Published:** 2019-11-27

**Authors:** Jaswinder Singh, Jack Hanson, Kuldip Paliwal, Yaoqi Zhou

**Affiliations:** 1 Signal Processing Laboratory, School of Engineering and Built Environment, Griffith University, Brisbane, QLD 4111 Australia; 2 Institute for Glycomics and School of Information and Communication Technology, Griffith University, Parklands Dr., Southport, QLD 4222 Australia

**Keywords:** Computational models, Machine learning

## Abstract

The majority of our human genome transcribes into noncoding RNAs with unknown structures and functions. Obtaining functional clues for noncoding RNAs requires accurate base-pairing or secondary-structure prediction. However, the performance of such predictions by current folding-based algorithms has been stagnated for more than a decade. Here, we propose the use of deep contextual learning for base-pair prediction including those noncanonical and non-nested (pseudoknot) base pairs stabilized by tertiary interactions. Since only $$<$$250 nonredundant, high-resolution RNA structures are available for model training, we utilize transfer learning from a model initially trained with a recent high-quality bpRNA dataset of $$> $$10,000 nonredundant RNAs made available through comparative analysis. The resulting method achieves large, statistically significant improvement in predicting all base pairs, noncanonical and non-nested base pairs in particular. The proposed method (SPOT-RNA), with a freely available server and standalone software, should be useful for improving RNA structure modeling, sequence alignment, and functional annotations.

## Abstract (web summary)

The limited availability of high-resolution 3D RNA structures for model training limits RNA secondary structure prediction. Here, the authors overcome this challenge by pre-training a DNN on a large set of predicted RNA structures and using transfer learning with high-resolution structures.

## Introduction

RNA secondary structure is represented by a list of the nucleotide bases paired by hydrogen bonding within its nucleotide sequence. Stacking these base pairs forms the scaffold driving the folding of RNA three-dimensional structures^1^. As a result, the knowledge of the RNA secondary structure is essential for modeling RNA structures and understanding their functional mechanisms. As such, many experimental methods have been developed to infer paired bases by using one-dimensional or multiple-dimensional probes, such as enzymes, chemicals, mutations, and cross-linking techniques coupled with next-generation sequencing^2,3^. However, precise base-pairing information at the resolution of single base pairs still requires high-resolution, three-dimensional RNA structures determined by X-ray crystallography, nuclear magnetic resonance (NMR), or cryogenic electron microscopy. With $<0.01 \%$ of 14 million noncoding RNAs collected in RNAcentral^4^ having experimentally determined structures^5^, it is highly desirable to develop accurate and cost-effective computational methods for direct prediction of RNA secondary structure from sequence.

Current RNA secondary-structure prediction methods can be classified into comparative sequence analysis and folding algorithms with thermodynamic, statistical, or probabilistic scoring schemes^6^. Comparative sequence analysis determines base pairs conserved among homologous sequences. These methods are highly accurate^7^ if a large number of homologous sequences are available and those sequences are manually aligned with expert knowledge. However, only a few thousand RNA families are known in Rfam^8^. As a result, the most commonly used approach for RNA secondary-structure prediction is to fold a single RNA sequence according to an appropriate scoring function. In this approach, RNA structure is divided into substructures such as loops and stems according to the nearest-neighbor model^9^. Dynamic programming algorithms are then employed for locating the global minimum or probabilistic structures from these substructures. The scoring parameters of each substructure can be obtained experimentally^10^ (e.g., RNAfold^11^, RNAstructure^12^, and RNAshapes^13^) or by machine learning (e.g., CONTRAfold^14^, CentroidFold^15^, and ContextFold^16^). However, the overall precision (the fraction of correctly predicted base pairs in all predicted base pairs) appears to have reached a “performance ceiling”^6^ at about 80$\%$^17,18^. This is in part because all existing methods ignore some or all base pairs that result from tertiary interactions^19^. These base pairs include lone (unstacked), pseudoknotted (non-nested), and noncanonical (not A–U, G–C, and G–U) base pairs as well as triplet interactions^19,20^. While some methods can predict RNA secondary structures with pseudoknots (e.g., pknotsRG^21^, Probknot^22^, IPknot^23^, and Knotty^24^) and others can predict noncanonical base pairs (e.g., MC-Fold^25^, MC-Fold-DP^26^, and CycleFold^27^), none of them can provide a computational prediction for both, not to mention lone base pairs and base triplets.

The work presented in this paper is inspired by a recent advancement in the direct prediction of protein contact maps from protein sequences by Raptor-X^28^ and SPOT-Contact^29^ with deep-learning neural network algorithms such as Residual Networks (ResNets)^30^ and two-dimensional Bidirectional Long Short-Term Memory cells (2D-BLSTMs)^31,32^. SPOT-Contact treats the entire protein “image” as context and used an ensemble of ultra-deep hybrid networks of ResNets coupled with 2D-BLSTMs for prediction. ResNets can capture contextual information from the whole sequence “image” at each layer and map the complex relationship between input and output. Also, 2D-BLSTMs proved very effective in propagating long-range sequence dependencies in protein structure prediction^29^ because of the ability of LSTM cells to remember the structural relationship between the residues that are far from each other in their sequence positions during training. Similar to protein contact map, a RNA secondary structure is a two-dimensional contact matrix, although its contacts are defined differently (hydrogen bonds for RNA base pairs and distance cutoff for protein contacts, respectively). However, unlike proteins, the small number of nonredundant RNA structures available in the Protein Data Bank (PDB)^5^ makes deep-learning methods unsuitable for direct single-sequence-based prediction of RNA secondary structure. As a result, machine-learning techniques are rarely utilized. To our knowledge, the only example is mxfold^33^ that employs a small-scale machine-learning algorithm (structured support vector machines) for RNA secondary-structure prediction. Its performance after combining with a thermodynamic model makes some improvement over folding-based techniques. However, mxfold is limited to canonical base pairs without accounting for pseudoknots.

Recently, a large database of more than 100,000 RNA sequences (bpRNA^34^) with automated annotation of secondary structure was released. While this database is large enough for us to employ deep-learning techniques, the annotated secondary structures from the comparative analysis may not be reliable at the single base-pair level. To overcome this limitation, we first employed bpRNA to train an ensemble of ResNets and LSTM networks, similar to the ensemble used by us for protein contact map prediction by SPOT-Contact^29^. We then further trained the large model with a small database of precise base pairs derived from high-resolution RNA structures. This transfer-learning technique^35^ is used successfully by us for identifying molecular recognition features in intrinsically disordered regions of proteins^36^. The resulting method, called SPOT-RNA, is a deep-learning technique for predicting all bases paired, regardless if they are associated with tertiary interactions. The new method provides more than 53$\%$, 47$\%$, and 10$\%$ improvement in F1 score for non-nested, noncanonical, and all base pairs, respectively, over the next-best method, compared with an independent test set of 62 high-resolution RNA structures by X-ray crystallography. The performance of SPOT-RNA is further confirmed by a separate test set of 39 RNA structures determined by NMR and 6 recently released nonredundant RNAs in PDB.

## Results

### Initial training by bpRNA

**Fig. 1:**
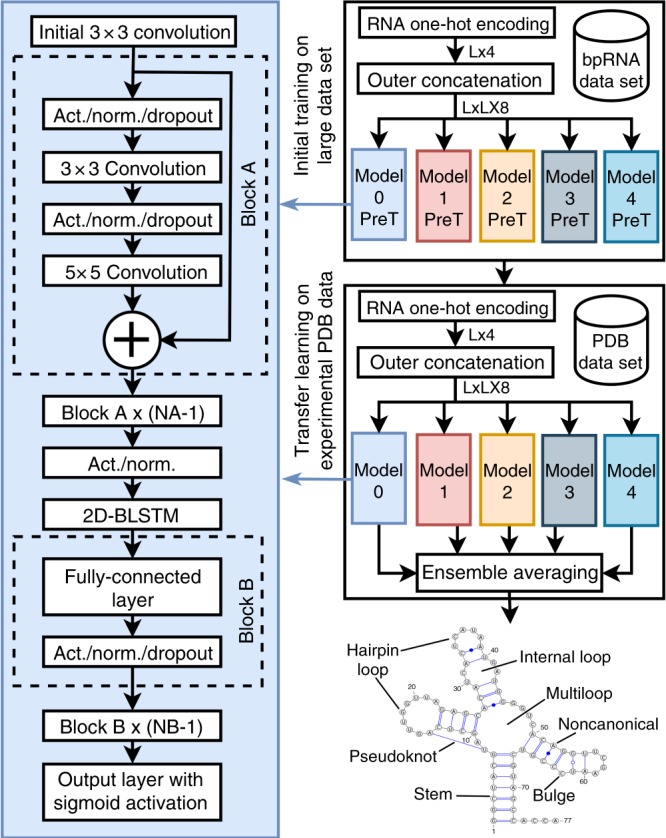
Generalized model architecture of SPOT-RNA. The network layout of the SPOT-RNA, where $L$ is the sequence length of a target RNA, Act. indicates the activation function, Norm. indicates the normalization function, and PreT indicates the pretrained (initial trained) models trained on the bpRNA dataset.

We trained our models of ResNets and LSTM networks by building a nonredundant set of RNA sequences with annotated secondary structure from bpRNA^34^ at 80$$\%$$ sequence-identity cutoff, which is the lowest sequence-identity cutoff allowed by the program CD-HIT-EST^37^ and has been employed previously by many studies for the same purpose^38,39^. This dataset of 13,419 RNAs after excluding those $$> $$80$$\%$$ sequence identities was further randomly divided into 10,814 RNAs for training (TR0), 1300 for validation (VL0), and 1,305 for an independent test (TS0). By using TR0 for training, VL0 for validation, and the single sequence (a one-hot vector of *L*x4) as the only input, we trained many two-dimensional deep-learning models with various combinations in the numbers and sizes of ResNets, BLSTM, and FC layers with a layout shown in Fig. 1. The performance of an ensemble of the best 5 models (validated by VL0 only) on VL0 and TS0 is shown in Table 1. Essentially the same performance with Matthews correlation coefficient (MCC) at 0.632 for VL0 and 0.629 for TS0 suggests the robustness of the ensemble trained. The F1 scores, the harmonic mean of precision, and sensitivity are also essentially the same between validation and test (0.629 vs. 0.626). Supplementary Table 1 further compared the performance of individual models to the ensemble. The MCC improves by 2$$\%$$ from 0.617 (the best single model) to 0.629 in TS0, confirming the usefulness of an ensemble to eliminate random prediction errors in individual models.

**Table 1:** Performance of SPOT-RNA on validation and test set after initial training, transfer learning, and direct training.

Method	Training set	Analysis set	MCC${}^{a}$	F1${}^{b}$	Precision	Sensitivity
Initial training	TR0	VL0	0.632	0.629	0.712	0.563
	TR0	TS0	0.629	0.626	0.709	0.560
	TR0	TS1	0.650	0.630	0.897	0.485
Transfer learning	TR1+VL1	TR1+VL1	0.701 (0.02${}^{c}$)	0.690 (0.02${}^{c}$)	0.853 (0.02${}^{c}$)	0.580 (0.03${}^{c}$)
	TR1+VL1	TS1	0.690 (0.02${}^{c}$)	0.687 (0.01${}^{c}$)	0.888 (0.02${}^{c}$)	0.562 (0.02${}^{c}$)
Direct training	TR1	VL1	0.583	0.546	0.854	0.401
	TR1	TS1	0.571	0.527	0.870	0.378

^a^Matthews correlation coefficient

^b^Harmonic mean of precision and sensitivity

^c^Standard deviation based on five-fold cross-validation

### Transfer learning with RNA structures

The models obtained from the bpRNA dataset were transferred to further train on base pairs derived from high-resolution nonredundant RNA structures with TR1 (training set), VL1 (validation set), and TS1 (test set) having 120, 30, and 67 RNAs, respectively. The TS1 set is independent of the training data (TR0 and TR1) as it was obtained by first filtering through CD-HIT-EST at the lowest allowed sequence-identity cutoff (80$\%$). To further remove potential homologies, we utilized BLAST-N^40^ against the training data (TR0 and TR1) with an e-value cutoff of 10. To examine the consistency of the models built, we performed 5-fold cross-validation by combining TR1 and VL1 datasets. The results of cross-validation on training data (TR1+VL1) and unseen TS1 for the ensemble of the same top 5 models are shown in Table 1. The minor fluctuations on 5-fold with MCC of 0.701$\pm$0.02 and F1 of 0.690$\pm$0.02 and small difference between 5-fold cross-validation and test set TS1 (0.701 vs. 0.690 for MCC) indicate the robustness of the models trained for the unseen data. Table 1 also shows that the direct application of the model trained by bpRNA leads to a reasonable but inferior performance on TS1 compared with the model after transfer learning. The improvement in MCC is 6$\%$ before (0.650) and after (0.690) transfer learning on TS1. Supplementary Tables 2 and 3 compare the result of the ensemble of models and five individual models for five-fold cross-validation (TR1+VL1) and independent test set (TS1), respectively. Significant improvement of the ensemble over the best single model is observed with 3$\%$ improvement in MCC for cross-validation and independent tests.

### Comparison between transfer learning and direct learning

**Fig. 2:**
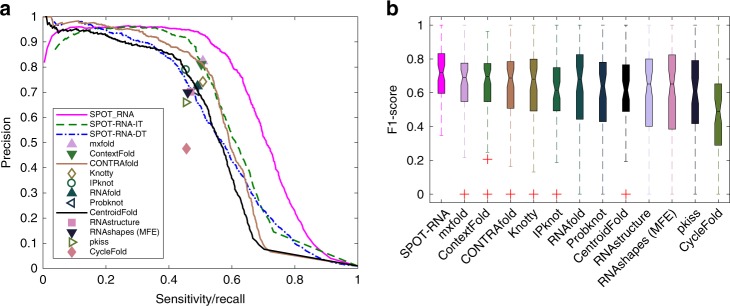
Performance comparison of SPOT-RNA with 12 other predictors by using PR curve and boxplot on the test set TS1. **a** Precision-recall curves on the independent test set TS1 by initial training (SPOT-RNA-IT, the green dashed line), direct training (SPOT-RNA-DT, the blue dot-dashed line), and transfer learning (SPOT-RNA, the solid magenta line). Precision and sensitivity results from ten currently used predictors are also shown as labeled with open symbols for the methods accounting for pseudoknots and filled symbols for the methods not accounting for pseudoknots. CONTRAfold and CentroidFold were also shown as curves (Gold and Black) because their methods provide predicted probabilities. **b** Distribution of F1 score for individual RNAs on the independent test set TS1 given by various methods as labeled. On each box, the central mark indicates the median, and the bottom and top edges of the box indicate the 25th and 75th percentiles, respectively. The outliers are plotted individually by using the “+” symbol.

To demonstrate the usefulness of transfer learning, we also perform the direct training of the 5 models with the same ensemble network architecture and hyperparameters (the number of layers, the depth of layers, the kernel size, the dilation factor, and the learning rate) on the structured RNA train set (TR1) and validated by VL1 and tested by TS1. The performance of the ensemble of five models by direct learning on VL1 and TS1 is shown in Table 1. Similar performance between validation and test with MCC = 0.583, 0.571, respectively, confirms the robustness of direct learning. However, this performance is substantially lower than that of transfer learning (21$\%$ reduction of the MCC value and 30$\%$ reduction in F1 score). This confirms the difficulty of direct learning with a small training dataset of TR1 and the need for using a large dataset (bpRNA) that can effectively utilize capabilities of deep-learning networks. Supplementary Table 4 further compared the performance of individual models with the ensemble by direct learning on TR1. Figure 2a compares the precision-recall (PR) curves given by initial training (SPOT-RNA-IT), direct training (SPOT-RNA-DT), and transfer learning (SPOT-RNA) on the independent test set TS1. The results are from a reduced TS1 (62 RNAs rather than 67) because some other methods shown in the same figure do not predict secondary structure for sequences with missing or invalid bases. Interestingly, direct training starts with 100$\%$ precision at very low sensitivity (recall), whereas both initial training and transfer learning have high but $<$100$\%$ precision at the lowest achievable sensitivities for the highest possible threshold that separates positive from negative prediction. This suggests that the existence of false positives in bpRNA “contaminated” the initial training. Nevertheless, the transfer learning achieves a respectable 93.2$\%$ precision at 50$\%$ recall. This indicates that the fraction of potential false positives in bpRNA is small.

### Comparison with other secondary-structure predictors

Figure 2a further compares precision/recall curves given by our transfer-learning ensemble model with 12 other available RNA secondary-structure predictors on independent test set TS1. Two predictors (CONTRAfold and CentroidFold) with probabilistic outputs are also represented by the PR curves with the remaining shown as a singular point. The performance of most existing methods is clustered around the sensitivity of 50$\%$ and precision of 67–83$\%$ (Table 2). By comparison, our method SPOT-RNA improves by 9$\%$ in MCC and more than 10$\%$ in F1 score over the next-best mxfold.

**Table 2:** Performance of all the predictors according to base-pair types on the test set TS1.

	All base pairs				Canonical only			Watson–Crick only			Wobble only		
	MCC${}^{a}$	F1${}^{b}$	Precision	Sensitivity	F1${}^{b}$	Precision	Sensitivity	F1${}^{b}$	Precision	Sensitivity	F1${}^{b}$	Precision	Sensitivity
SPOT-RNA	0.700	0.690	0.849	0.582	0.773	0.858	0.703	0.790	0.857	0.733	0.592	0.865	0.450
mxfold	0.644	0.628	0.824	0.508	0.728	0.824	0.652	0.749	0.830	0.682	0.519	0.747	0.398
ContextFold	0.636	0.621	0.811	0.503	0.719	0.811	0.646	0.737	0.822	0.668	0.554	0.693	0.462
CONTRAfold	0.621	0.611	0.765	0.508	0.704	0.765	0.652	0.724	0.778	0.677	0.517	0.630	0.439
Knotty	0.611	0.603	0.742	0.508	0.694	0.742	0.652	0.713	0.755	0.676	0.519	0.611	0.450
IPknot	0.596	0.576	0.789	0.454	0.671	0.789	0.583	0.690	0.799	0.608	0.483	0.681	0.374
RNAfold	0.593	0.585	0.724	0.491	0.674	0.724	0.630	0.696	0.742	0.655	0.478	0.554	0.421
ProbKnot	0.582	0.576	0.705	0.486	0.662	0.705	0.624	0.684	0.725	0.648	0.466	0.522	0.421
CentroidFold	0.577	0.569	0.706	0.477	0.656	0.706	0.612	0.675	0.719	0.636	0.476	0.569	0.409
RNAstructure	0.570	0.562	0.702	0.469	0.648	0.702	0.602	0.670	0.719	0.627	0.451	0.532	0.392
RNAshapes	0.564	0.555	0.699	0.460	0.640	0.699	0.591	0.661	0.716	0.614	0.455	0.531	0.398
pkiss	0.543	0.538	0.660	0.454	0.619	0.660	0.582	0.643	0.682	0.608	0.403	0.453	0.363
CycleFold	0.461	0.466	0.476	0.456	0.546	0.551	0.540	0.565	0.566	0.564	0.368	0.403	0.339

^a^Matthews correlation coefficient

^b^Harmonic mean of precision and sensitivity

The results presented in Fig. 2a are the overall performance at the base-pair level. Figure 2b shows the distribution of the F1 score among individual RNAs in terms of median, 25th, and 75th percentiles. SPOT-RNA has the highest median F1 score along with the highest F1 score (0.348) for the worst-performing RNA, compared with nearly 0 for all other methods. This highlights the highly stable performance of SPOT-RNA, relative to all other folding-based techniques, including mxfold, which mixes thermodynamic and machine-learning models. The difference between SPOT-RNA and the next-best mxfold on TS1 is statistically significant with *P* value $<$ 0.006 obtained through a paired *t* test. Also, we calculated the ensemble defect (see the “Methods” section) from the predicted base-pair probabilities for SPOT-RNA, CONTRAfold, and CentroidFold on TS1. The ensemble defect metric describes the deviation of probabilistic structural ensembles from their corresponding native RNA secondary structure, where 0 represents a perfect prediction. The ensemble defect for SPOT-RNA was 0.19 as compared with 0.24 and 0.25 for CONTRAfold and CentroidFold, respectively, showing that the structural ensemble predicted by SPOT-RNA is more similar to target structures in comparison with the other two predictors.

Our method was trained for RNAs with a maximum length of 500 nucleotides, due to hardware limitations. It is of interest to determine how our method performs in terms of size dependence. As the maximum sequence length in TS1 was 189, therefore, we added 32 RNAs of sequence length from 298 to 1500 to TS1 by relaxing the resolution requirement to 4 Å and including RNA chains complexed with other RNAs (but ignored inter-RNA base pairs). The reason for relaxing the resolution to 4 Å and including RNA chains complexed with other RNAs because there were not many high-resolution and single-chain long RNAs in PDB. Supplementary Fig. 1 compares the F1 score of each RNA given by SPOT-RNA with that from the next-best mxfold as a function of the length of RNAs. There is a trend of lower performance for a longer RNA chain for both methods as expected. SPOT-RNA consistently outperforms mxfold within 500 nucleotides that our method was trained on. Supplementary Fig. 1 also shows that mxfold performs better with an average of F1 score at 0.50, compared with 0.35 by SPOT-RNA on 21 long RNAs (*L* $$> $$ 1000). We found that the poor performance of SPOT-RNA is mainly because of the failure of SPOT-RNA to capture ultra long-distance pairs with sequence separation $$> $$300. This failure is caused by the limited long RNA data in training. By comparison, the thermodynamic algorithm in mxfold can locate the global minimum regardless of the distance between sequence positions of the base pairs.

The above comparison may be biased toward our method because almost all other methods compared can only predict canonical base pairs, which include Watson–Crick (A–U and G–C) pairs and Wobble pairs (G–U). To address this potential bias, Table 2 further compares the performance of SPOT-RNA with others on canonical pairs, Watson–Crick pairs (A–U and G–C pairs), and Wobble pairs (G–U), separately on TS1. Indeed, all methods have a performance boost when noncanonical pairs are excluded from performance measurement. SPOT-RNA continues to have the best performance with 6$\%$ improvement in F1 score for canonical pairs and Watson–Crick pairs over the next-best mxfold and 7$\%$ improvement for Wobble pairs over the next-best ContextFold. mxfold does not perform as well in predicting Wobble pairs and is only the fourth best.

Base pairs associated with pseudoknots are challenging for both folding-based and machine-learning-based approaches because they are often associated with tertiary interactions that are difficult to predict. To make a direct comparison in the capability of predicting base pairs in pseudoknots, we define pseudoknot pairs as the minimum number of base pairs that can be removed to result in a pseudoknot-free secondary structure. The program bpRNA^34^ (available at https://github.com/hendrixlab/bpRNA) was used to obtain base pairs in pseudoknots from both native and predicted secondary structures. Table 3 compares the performance of SPOT-RNA with all 12 other methods regardless if they can handle pseudoknots or not for those 40 RNAs with at least one pseudoknot in the independent test TS1. As none of the other methods predict multiplets, we ignore the base pairs associated with the multiplets in the analysis. mxfold remains the second best behind SPOT-RNA although it is unable to predict pseudoknots, due to the number of base pairs in pseudoknots accounting for only 10$\%$ of all base pairs (see Supplementary Table 7). Table 3 shows that all methods perform poorly with F1 score < 0.3 for base pairs associated with pseudoknots. Despite the challenging nature of this problem, SPOT-RNA makes a substantial improvement over the next-best (pkiss) by 52$\%$ in F1 score.

**Table 3:** Performance of all the predictors on 40 pseudoknot RNAs in the test set TS1.

	All Base Pairs				Base Pairs in Pseudoknots			Base Pair not in Pseudoknots		
	MCC${}^{a}$	F1${}^{b}$	Precision	Sensitivity	F1${}^{b}$	Precision	Sensitivity	F1${}^{b}$	Precision	Sensitivity
SPOT-RNA	0.769	0.764	0.875	0.679	0.239	0.550	0.153	0.797	0.872	0.734
mxfold	0.687	0.682	0.797	0.595	0.000	0.000	0.000	0.714	0.780	0.659
ContextFold	0.686	0.680	0.797	0.594	0.000	0.000	0.000	0.714	0.781	0.658
CONTRAfold	0.659	0.658	0.735	0.595	0.000	0.000	0.000	0.688	0.719	0.659
Knotty	0.678	0.678	0.740	0.625	0.108	0.134	0.090	0.707	0.761	0.660
IPknot	0.638	0.629	0.769	0.533	0.131	0.458	0.076	0.664	0.768	0.585
RNAfold	0.605	0.606	0.666	0.555	0.000	0.000	0.000	0.646	0.666	0.628
ProbKnot	0.610	0.611	0.669	0.562	0.118	0.256	0.076	0.632	0.663	0.603
CentroidFold	0.616	0.616	0.682	0.562	0.000	0.000	0.000	0.644	0.668	0.621
RNAstructure	0.585	0.584	0.650	0.531	0.000	0.000	0.000	0.621	0.647	0.598
RNAshapes	0.569	0.568	0.639	0.512	0.000	0.000	0.000	0.591	0.622	0.563
pkiss	0.564	0.565	0.619	0.520	0.157	0.180	0.139	0.566	0.616	0.523
CycleFold	0.455	0.458	0.423	0.499	0.000	0.000	0.000	0.482	0.422	0.563

^a^Matthews correlation coefficient

^b^Harmonic mean of precision and sensitivity

Noncanonical pairs, triplets, and lone base pairs are also associated with tertiary interactions other than pseudoknots. Here, lone base pairs refer to a single base pair without neighboring base pairs (i.e., [*i*, *j*] in the absence of [*i* − 1, *j* + 1] and [*i* + 1, *j* − 1]). Triplets refer to the rare occasion of one base forming base pairs with two other bases. As shown in Supplementary Table 5, SPOT-RNA makes a 47$\%$ improvement in F1 score for predicting noncanonical base pairs over CycleFold. Although the sensitivity of prediction given by SPOT-RNA is low (15.4$\%$), the precision is high at 73.2$\%$. Very low performance for triplets and lone pairs (F1 score $<$ 0.2) is observed.

Secondary structure of RNAs is characterized by structural motifs in their layout. For each native or predicted secondary structure, the secondary-structure motif was classified by program bpRNA^34^. The performance in predicting bases in different secondary structural motifs by different methods is shown in Table 4. According to the F1 score, SPOT-RNA makes the best prediction in stem base pairs (6$\%$ improvement over the next best), hairpin loop nucleotide (8$\%$ improvement), and bulge nucleotide (11$\%$ improvement), although it performs slightly worse than CONTRAfold in multiloop (by 2$\%$). mxfold is best for internal loop prediction over the second-best predictor Knotty by 18$\%$. To demonstrate the SPOT-RNA’s ability to predict tertiary interactions along with canonical base pairs, Supplementary Figs. 2 and 3 show two examples (riboswitch^41^ and t-RNA^42^) from TS1 with one high performance and one average performance, respectively. For both the examples, SPOT-RNA is able to predict noncanonical base pairs (in green), pseudoknot base pairs, and lone pair (in blue), while mxfold and IPknot remain unsuccessful to predict noncanonical and pseudoknot base pairs.

**Table 4:** Performance of all the predictors on secondary-structure motifs on the test set TS1.

	Stem(F1^*a*^)	Stem (PR)	Stem (SN)	Hairpin loop (F1^*a*^)	Hairpin loop (PR)	Hairpin loop (SN)	Bulge (F1^*a*^)	Bulge (PR)	Bulge (SN)	Internal loop (F1^*a*^)	Internal loop (PR)	Internal loop (SN)	Multiloop (F1^*a*^)	Multiloop (PR)	Multiloop (SN)
SPOT-RNA	0.762	0.841	0.697	0.686	0.625	0.761	0.369	0.508	0.289	0.266	0.239	0.300	0.562	0.503	0.638
mxfold	0.717	0.769	0.671	0.625	0.525	0.771	0.213	0.360	0.152	0.329	0.270	0.422	0.526	0.465	0.607
ContextFold	0.706	0.755	0.663	0.633	0.513	0.825	0.286	0.539	0.194	0.214	0.170	0.289	0.574	0.544	0.607
CONTRAfold	0.688	0.705	0.671	0.624	0.553	0.715	0.331	0.378	0.294	0.279	0.241	0.331	0.469	0.587	0.391
Knotty	0.670	0.739	0.613	0.600	0.493	0.766	0.295	0.421	0.227	0.279	0.238	0.338	0.549	0.649	0.476
IPknot	0.665	0.754	0.595	0.602	0.510	0.735	0.201	0.474	0.128	0.218	0.202	0.236	0.417	0.339	0.542
RNAfold	0.671	0.686	0.657	0.617	0.539	0.722	0.313	0.500	0.227	0.270	0.218	0.354	0.514	0.555	0.478
ProbKnot	0.625	0.661	0.592	0.571	0.480	0.704	0.276	0.377	0.218	0.209	0.187	0.236	0.481	0.492	0.470
CentroidFold	0.646	0.662	0.632	0.579	0.467	0.761	0.293	0.395	0.232	0.179	0.211	0.156	0.433	0.379	0.506
RNAstructure	0.646	0.665	0.629	0.596	0.508	0.720	0.300	0.440	0.227	0.238	0.204	0.285	0.478	0.546	0.424
RNAshapes	0.627	0.650	0.605	0.574	0.507	0.663	0.310	0.432	0.242	0.238	0.193	0.308	0.433	0.507	0.378
pkiss	0.618	0.684	0.565	0.532	0.449	0.655	0.253	0.457	0.175	0.229	0.183	0.304	0.406	0.494	0.344
CycleFold	0.496	0.431	0.584	0.437	0.564	0.357	0.277	0.333	0.237	0.000	0.000	0.000	0.367	0.374	0.360

${}^{a}$Harmonic mean of precision (PR) and sensitivity (SN)

**Fig. 3:**
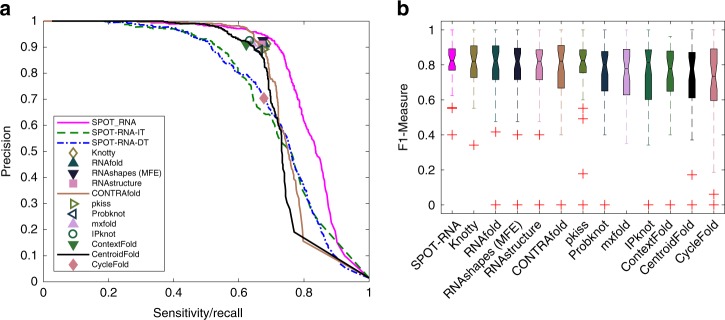
Performance comparison of SPOT-RNA with 12 other predictors by using PR curve and boxplot on the test set TS2. **a** Precision-recall curves on the independent test set TS2 by various methods as in Fig. 2a labeled. **b** Distribution of F1 score for individual RNAs on the independent test set TS2 given by various methods as in Fig. 2b labeled.

To further confirm the performance of SPOT-RNA, we compiled another test set (TS2) with 39 RNA structures solved by NMR. As with TS1, TS2 was made nonredundant to our training data by using CD-HIT-EST and BLAST-N. Figure 3a compares precision-recall curves given by SPOT-RNA with 12 other RNA secondary-structure predictors on the test set TS2. SPOT-RNA outperformed all other predictors on this test set (Supplementary Table 6). Furthermore, Fig. 3b shows the distribution of the F1 score among individual RNAs in terms of median, 25th, and 75th percentiles. SPOT-RNA achieved the highest median F1 score with the least fluctuation although the difference between SPOT-RNA and the next-best (Knotty this time) on individual RNAs (shown in Supplementary Fig. 4) is not significant with *P* value $<$ 0.16 obtained through a paired *t* test. Ensemble defect on TS2 is the smallest by SPOT-RNA (0.14 for SPOT-RNA as compared with 0.18 and 0.19 by CentroidFold and CONTRAfold, respectively). Here, we did not compare the performance in pseudoknots because the number of base pairs in pseudoknots (a total of 21) in this dataset is too small to make statistically meaningful comparison.

**Fig. 4:**
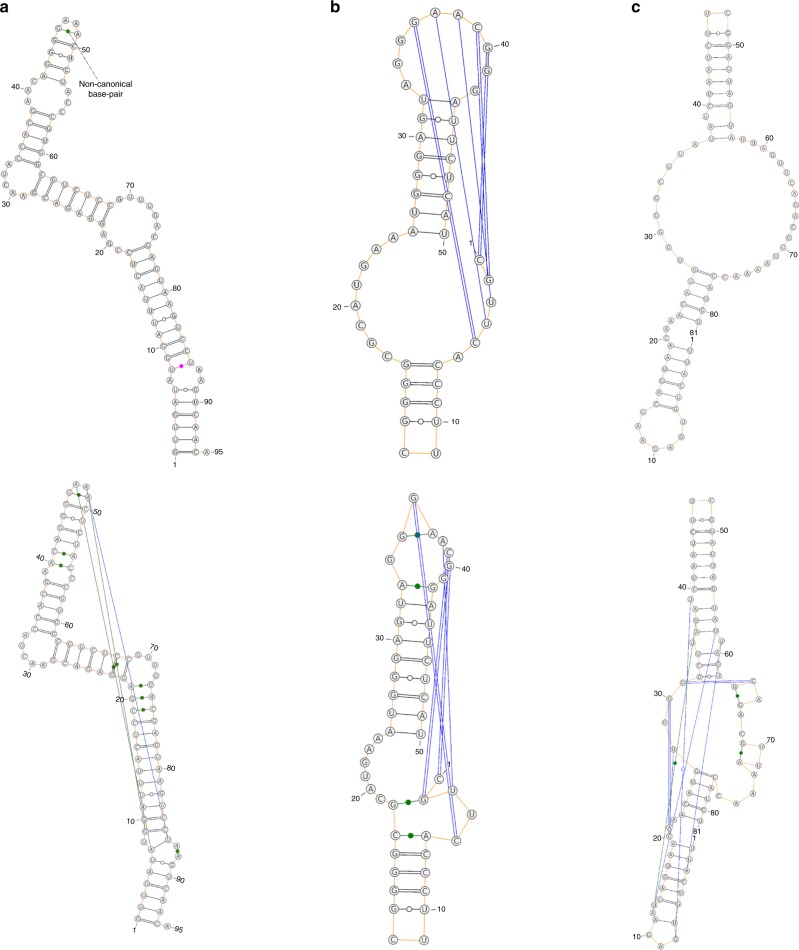
Comparison of SPOT-RNA prediction with the native structure of a Synthetic Construct, Glutamine II Riboswitch, and Hatchet Ribozyme. The secondary structure of a synthetic construct RNA (chain H in PDB ID 6dvk), the Glutamine II Riboswitch RNA (chain A in PDB ID 6qn3), and Synthetic Construct Hatchet Ribozyme (chain U in PDB ID 6jq6) represented by 2D diagram with canonical base pair (BP) in black color, noncanonical BP in green color, pseduoknot BP and lone pair in blue color, and wrongly predicted BP in magenta color: **a** predicted structure by SPOT-RNA (at top), with 97$\%$ precision and 77$\%$ sensitivity, as compared with the native structure (at bottom) for the Synthetic Construct RNA, **b** the predicted structure by SPOT-RNA (at top) with 100$\%$ precision and 81$\%$ sensitivity, as compared with the native structure (at bottom) for the Riboswitch, **c** the predicted structure by SPOT-RNA (at top) with 100$\%$ precision and 59$\%$ sensitivity, as compared with the native structure (at bottom) for the synthetic construct Hatchet ribozyme.

In addition, we found a total of 6 RNAs with recently solved structures (after March 9, 2019) that are not redundant according to CD-HIT-EST and BLAST-N to our training sets (TR0 and TR1) and test sets (TS1 and TS2). The prediction for a synthetic construct RNA (released on 26 June 2019, chain H in PDB ID 6dvk)^43^ was compared with the native structure in Fig. 4a. For this synthetic RNA, SPOT-RNA yields a structural topology very similar to the native secondary structure with F1 score of 0.85, precision of 97$\%$, and sensitivity of 77$\%$. In particular, SPOT-RNA captures one noncanonical base pair between G46 and A49 correctly but missed others in pseudoknots. The SPOT-RNA predictions of Glutamine II Riboswitch (chain A in PDB ID 6qn3, released on June 12, 2019)^44^ and Synthetic Construct Hatchet Ribozyme (chain U in PDB ID 6jq6, released on June 12, 2019)^45^ are compared with their respective native secondary structure in Fig. 4b, c, respectively. For these two RNAs, experimental evidence suggests strand swapping in dimerization^44,45^. Thus, their monomeric native structures are obtained by replacing the swapped stand by its original stand. SPOT-RNA is able to predict both the stems and pseudoknot (in Blue) with an overall F1 score of 0.90 for Glutamine II Riboswitch. For Hatchet Ribozyme, SPOT-RNA is able to predict native-like structure with F1 score of 0.74 although it has missed noncanonical and pseudoknot base pairs.

**Fig. 5:**
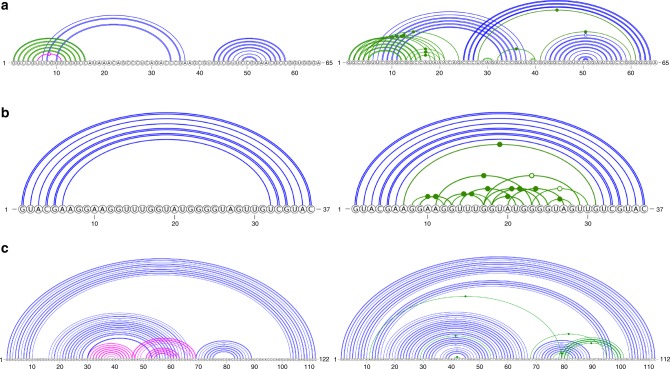
Comparison of SPOT-RNA prediction with the native structure of a Pistol Ribozyme, Mango aptamer, and Adenovirus Virus-associated RNA. The secondary structure of a Pistol Ribozyme (chain A and B in PDB ID 6r47), the Mango Aptamer (chain B in PDB ID 6e8u), and the adenovirus virus-associated RNA (chain C in PDB ID 6ol3) represented by arc diagrams with canonical base pair (BP) in blue color, noncanonical, pseduoknot BP and lone pair in green color, and wrongly predicted BP in magenta color: **a** predicted structure by SPOT-RNA (on left), with 93$\%$ precision and 41$\%$ sensitivity, as compared with the native structure (on right) for the Pistol Ribozyme, **b** the predicted structure by SPOT-RNA (on left) with 100$\%$ precision and 26$\%$ sensitivity, as compared with the native structure (on right) for the Mango aptamer, **c** the predicted structure by SPOT-RNA (on left) with 66$\%$ precision and 60$\%$ sensitivity, as compared with the native structure (on right) for the adenovirus virus-associated RNA.

Three other RNAs are Pistol Ribozyme (chain A and B in PDB ID 6r47, released on July 3, 2019)^46^, Mango Aptamer (chain B in PDB ID 6e8u, released on April 17, 2019)^47^, and Adenovirus Virus-associated RNA (chain C in PDB ID 6ol3, released on July 3, 2019)^48^. SPOT-RNA achieves F1 score of 0.57, 0.41, and 0.63 on Pistol Ribozyme, Mango Aptamer, and adenovirus virus-associated RNA, respectively. For this level of performance, it is more illustrative to show a one-dimensional representation of RNA secondary structure (Fig. 5a–c). The figures show that the relatively poor performance of Pistol Ribozyme and Mango Aptamer RNAs is in part due to the uncommon existence of a large number of noncanonical base pairs (in Green). For adenovirus virus-associated RNA (VA-I), SPOT-RNA’s prediction is poor. It contains three false-positive stems with falsely predicted pseudoknots (Fig. 5c).

**Fig. 6:**
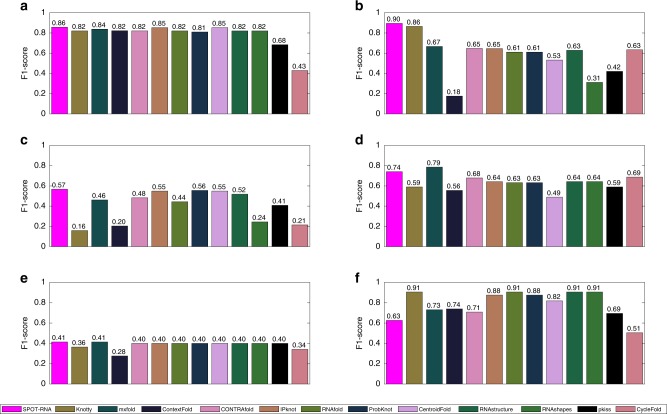
Performance comparison of all predictors on 6 recently released (after March 9, 2019) crystal structures. **a** F1 score of predicted structure on a synthetic construct RNA (chain H in PDB ID 6dvk), **b** F1 score of predicted structure on the Glutamine II Riboswitch RNA (chain A in PDB ID 6qn3), **c** F1 score of predicted structure on a synthetic construct Hatchet Ribozyme (chain U in PDB ID 6jq6), **d** F1 score of predicted structure on a Pistol Ribozyme (chain A & B in PDB ID 6r47), **e** F1 score of predicted structure on the Mango Aptamer (chain B in PDB ID 6e8u), **f** F1 score of predicted structure on the adenovirus virus-associated RNA (chain C in PDB ID 6ol3).

Performance comparison on these 6 RNAs with 12 other secondary-structure predictors is shown in Fig. 6. SPOT-RNA outperforms all other predictors on Synthetic Construct RNA (Fig. 6a), Glutamine II Riboswitch (Fig. 6b), and Pistol Ribozyme (Fig. 6c). It is the co-first (same as mxfold) in Mango Aptamer (Fig. 6e) and the second best (behind mxfold only) in Hatchet Ribozyme (Fig. 6d). However, it did not do well on adenovirus virus-associated RNA (Fig. 6f), which was part of RNA puzzle-2017, when compared with other methods. This poor prediction compared with other methods is likely because this densely contacted, base-pairing network without pseudoknots (except those due to noncanonical base pairs) is most suitable for folding-based algorithms that maximize the number of stacked canonical base pairs.

## Discussion

This work developed RNA secondary-structure prediction method purely based on deep neural network learning from a single RNA sequence. Because only a small number of high-resolution RNA structures are available, deep-learning models have to be first trained by using a large database of RNA secondary structures (bpRNA) annotated according to comparative analysis, followed by transfer learning to the precise secondary structures derived from 3D structures. Although the slightly noisy data in bpRNA lead to an upbound around 96$\%$ for the precision (Fig. 2a), the model generated from transfer learning yields a substantial improvement (30$\%$ in F1 score) over the model based on direct learning TS1. Without the need for folding-based optimization, the transfer-learning model yields a method that can predict not only canonical base pairs but also those base pairs often associated with tertiary interactions, including pseudoknots, lone, and noncanonical base pairs. By comparing with 12 current secondary-structure prediction techniques by using the independent test of 62 high-resolution X-ray structures of RNAs, the method (SPOT-RNA) achieved 93$\%$ in precision, which is a 13$\%$ improvement over the second-best method mxfold when the sensitivity for SPOT-RNA is set to 50.8$\%$ as in mxfold.

One advantage of a pure machine-learning approach is that all base pairs can be trained and predicted, regardless if it is associated with local or nonlocal (tertiary) interactions. By comparison, a folding-based method has to have accurate energetic parameters to capture noncanonical base pairs and sophisticated algorithms for a global minimum search to account for pseudoknots. SPOT-RNA represents a significant advancement in predicting noncanonical base pairs. Its F1 score improves over CycleFold by 47$\%$ from 17$\%$ to 26$\%$ although both methods have a low sensitivity at about 16$\%$ (Supplementary Table 5). SPOT-RNA can also achieve the best prediction of base pairs in pseudoknots although the performance of all methods remains low with an F1 score of 0.239 for SPOT-RNA and 0.157 for the next-best (pkiss, Table 3). This is mainly because the number of base pairs in pseudoknots is low in the structural datasets (an average of 3–4 base pairs per pseudoknot RNA in TS1, see Supplementary Table 7). Moreover, a long stem of many stacked base pairs is easier to learn and predict than a few nonlocal base pairs in pseudoknot. As a reference for future method development, we also examined the ability of SPOT-RNA to capture triple interactions: one base paired with two other bases. Both precision and sensitivity are low (12$\%$ and 7$\%$, respectively, Supplementary Table 5). This is mainly because there is a lack of data on base triples in bpRNA for pretraining and the number of both triplets and quartets is only 1194 in the structural training set TR1.

To further confirm the performance, SPOT-RNA was applied to 39 RNA structures determined by NMR (TS2). Unlike X-ray structures, structures determined by NMRs resulted from minimization of experimental distance-based constraints. These 39 NMR structures, smaller with average length of 51 nucleotides, have only a total of 21 base pairs in pseudoknots. As a result, they are much easier to predict for all methods (MCC $$<$$ 0.7 except SPOT-RNA for TS1 but $$> $$0.74 for most methods in TS2). Despite of this, SPOT-RNA continues to have the best performance (Fig. 3, Supplementary Table 6, and Supplementary Fig. 4) as compared with other 12 predictors. Furthermore, the performance of SPOT-RNA was tested on 6 recently released nonredundant (to TR0 and TR1) RNAs in PDB. SPOT-RNA performs the best or the same as the best in 4 and the second best in 1 of the 6 RNAs (Fig. 6).

One limitation of SPOT-RNA is that it was trained by RNAs shorter than 500 nucleotides due to our hardware limitation. Within 500 nucleotides, SPOT-RNA provides a consistent improvement over existing techniques (Supplementary Fig. 1). However, for really long RNA chains ($$> $$1000), a purely machine-learning-based technique is not as accurate as some of the folding-algorithm-based methods such as mxfold as shown in Supplementary Fig. 1. The lack of training for long RNAs is the main reason. Currently, even if there is no hardware limitation, the number of high-resolution RNA structures with $$> $$500 nucleotides in PDB structures are too few to provide adequate training. Thus, at this stage, SPOT-RNA is most suitable for RNA length of $$<$$500.

In addition to prediction accuracy, high computational efficiency is necessary for RNA secondary-structure prediction because genome-scale studies are often needed. We found that the CPU time for predicting all 62 RNAs in the test set TS1 on a single thread of 32-core Intel Xenon(R) E5-2630v4 CPU is 540 s, which is faster than Knotty (2800 s) but slower than IPknot (1.2 s), ProbKnot (13 s), and pkiss (112 s). However, our distributed version can be easily run on multiple CPU threads or on GPUs. For example, by running SPOT-RNA on a single Nvidia GTX TITAN X GPU, the computation time for predicting all 62 RNAs would be reduced to 39 s. Thus, SPOT-RNA can feasibly be used for genome-scale studies.

This work has used a single RNA sequence as the only input. It is quite remarkable that relying on a single sequence alone can obtain a more accurate method than existing folding methods in secondary-structure prediction. For protein contact map prediction, evolution profiles generated from PSIBLAST^40^ and HHblits^49^ as well as direct coupling analysis among homologous sequences^50^ are the key input vectors responsible for the recent improvement in highly accurate prediction. Thus, one expects that a similar evolution-derived sequence profile generated from BLAST-N and direct/evolution-coupling analysis would further improve secondary-structure prediction for nonlocal base pairs in long RNAs, in particular. Indeed, recently, we have shown that using evolution-derived sequence profiles significantly improves the accuracy of predicting RNA solvent accessibility and flexibility^38,39^. For example, the correlation coefficient between predicted and actual solvent accessibility increases from 0.54 to 0.63 if a single sequence is replaced by a sequence profile from BLAST-N^38^. However, the generation of sequence profiles and evolution coupling is computationally time consuming. The resulting improvement (or lack of improvement) is strongly depending on the number of homologous sequences available in current RNA sequence databases. If the number of homologous sequences is too low (which is true for most RNAs), it may introduce more noise than the signal to prediction as demonstrated in protein secondary structure and intrinsic disorder prediction^51,52^. Moreover, synthetic RNAs will not have any homologous sequences. Thus, we present the method with single-sequence information as input in this study. Using sequence profiles and evolutionary coupling as input for RNA secondary-structure prediction is working in progress.

Another possible method for further improving SPOT-RNA is to employ the predicted probability as a restraint for folding with an appropriate scoring function. Such a dual-approach method will likely improve SPOT-RNA as folding optimization may have a better capability to capture nonlocal interactions between WC pairs for long RNAs, in particular as shown in Supplementary Fig. 1. However, a simple integration may not yield a large improvement for shorter chains ($<$500). In mxfold, combining machine-learning and thermodynamic models leads to 0.6$\%$ in one test set and 5$\%$ in another test set^33^. Moreover, most thermodynamic methods simply ignore noncanonical base pairs and many do not even account for pseudoknots. mxfold, for example, employs a pseudoknot-free thermodynamic method to combine with its machine-learning model. Thus, balancing the performance for canonical, noncanonical, and pseudoknots will require a careful selection of appropriate scoring schemes. A simple integration may lead to high performance in one type of base pair at the expense of other types of base pairs. Nevertheless, we found that if we simply keep only the base pair with the highest predicted probability in predicted triple interactions, SPOT-RNA would be improved by another 3$\%$ in F1 score (from 0.69 to 0.71 in TS1), confirming that there is some room for improvement. We will defer this for future studies.

The significantly improved performance in secondary-structure prediction should allow large improvement in modeling RNA 3D structures. This is because the method predicts not only canonical base pairs but also provides important tertiary contacts of noncanonical and non-nested base pairs. Thus, it can serve as a more accurate, quasi-three-dimensional frame to enable correct folding into the right RNA tertiary structure. The usefulness of 2D structure prediction for 3D structure modeling has been demonstrated in RNA Puzzles (blind RNA structure prediction)^53^. Moreover, improvement in predicting secondary structural motifs (stems, loops, and bulges, see Table 4) would allow better functional inference^54,55^, sequence alignment^56^, and RNA inhibitor design^57^. The method and datasets are available as a server and stand-alone software publicly at http://sparks-lab.org/jaswinder/server/SPOT-RNA/and https://github.com/jaswindersingh2/SPOT-RNA/.

## Methods

### Datasets

The datasets for initial training were obtained from bpRNA-1m (Version 1.0)^34^, which consists of 102,348 RNA sequences with annotated secondary structure. Sequences with sequence similarity of more than 80% were removed by using CD-HIT-EST^37^. About 80$\%$ sequence-identity cutoff was the lowest cutoff allowed by CD-HIT-EST and has been used previously as an RNA nonredundancy cutoff^38,39^. After removing sequence similarity, 14,565 sequences remained. RNA sequences with RNA structures from the PDB^5^ available in this dataset were also removed as we prepared separate datasets based on RNAs with PDB structure only^5^. Moreover, due to hardware limitations for training on long sequences, the maximum sequence length was restricted to 500. After preprocessing, this dataset contains 13,419 sequences. These sequences were randomly split into 10,814 RNAs for training (TR0), 1300 for validation (VL0), and 1,305 for independent test (TS0). Supplementary Table 7 shows the number of RNA sequences and their Watson–Crick (A–U and G–C), Wobble (G–U), and noncanonical base-pair count as well as the number of base pairs associated with pseudoknots. The average sequence lengths in TR0, VL0, and TS0 are all roughly 130. Here, base pairs associated with pseudoknots are defined as the minimum number of base pairs that can be removed to result in a pseudoknot-free secondary structure. Pseudoknot labels were generated by using software bpRNA^34^ (available at https://github.com/hendrixlab/bpRNA).

The datasets for transfer learning were obtained by downloading high-resolution ($<$3.5 Å) RNAs from PDB on March 2, 2019^5^. Sequences with similarity of more than 80$\%$ among these sequences were removed with CD-HIT-EST^37^. After removing sequence similarity, only 226 sequences remained. These sequences were randomly split into 120, 30, and 76 RNAs for training (TR1), validation (VL1), and independent test (TS1), respectively. Furthermore, any sequence in TS1 having sequence similarity of more than 80$\%$ with TR0 was also removed, which reduced TS1 to 69 RNAs. As CD-HIT-EST can only remove sequences with similarity more than 80$\%$, we employed BLAST-N^40^ to further remove potential sequence homologies with training data with a large e-value cutoff of 10. This procedure further decreased TS1 from 69 to 67 RNAs.

To further benchmark RNA secondary-structure predictors, we employed 641 RNA structures solved by NMR. Using CD-HIT-EST with 80$\%$ identity cutoff followed by BLAST-N with *e*-value cutoff of 10 against TR0, TR1, and TS1, we obtained 39 NMR-solved structures as TS2.

The secondary structure of all the PDB sets was derived from their respective structures by using DSSR^58^ software. For NMR- solved structures, model 1 structure was used as it is considered as the most reliable structure among all. The numbers of canonical, noncanonical, and pseudoknot base pairs, and base multiplets (triplets and quartets) for all the sets are listed in Supplementary Table 7. These datasets along with annotated secondary structure are publicly available at http://sparks-lab.org/jaswinder/server/SPOT-RNA/ and https://github.com/jaswindersingh2/SPOT-RNA.

### RNA secondary-structure types

For the classification of different RNA secondary-structure types, we used the same definitions as previously used by bpRNA^34^. A stem is defined as a region of uninterrupted base pairs, with no intervening loops or bulge. A hairpin loop is a sequence of unpaired nucleotides with both ends meeting at the two strands of a stem region. An internal loop is defined as two unpaired strands flanked by closing base pairs on both sides. A bulge is a special case of the internal loop where one of the strands is of length zero. A multiloop consists of a cycle of more than two unpaired strands, connected by stems. The distribution of different secondary-structure types in TR1, VL1, and TS1 (excluding multiplet base pairs) is shown in Supplementary Table 8. These secondary-structure classifications were obtained by using a secondary-structure analysis program bpRNA^34^.

### Deep neural networks

We employed an ensemble of deep-learning neural networks for pretraining. The ensemble is made of 5 top-ranked models based on their performance on VL0 with the architecture shown in Fig. 1, similar to what was used previously for protein contact prediction in SPOT-Contact^29^.

The architecture of each model consists of ResNet blocks followed by a 2D-BLSTM layer and a fully connected (FC) block. An initial convolution layer for pre-activation was used before our ResNet blocks as proposed in He et al.^30^. The initial convolution layer is followed by ${N}_{A}$ ResNet blocks (Block A in Fig. 1). Each ResNet block consists of two convolutional layers with a kernel size of $3\times 3$ and $5\times 5$, respectively, and a depth of ${D}_{\rm{RES}}$. The exponential linear units (ELU)^59^ activation function and the layer normalization technique^60^ were used. A dropout rate of 25$\%$ was used before each convolution layer to avoid overfitting during training^61^. In some models, we used dilated convolutions that are reported to better learn longer-range dependencies^62^. For the dilated convolutional layers, the dilation factor was set to ${2}^{i \% n}$, where $i$ is the depth of the convolution layer, $n$ is a fixed scalar, and $\%$ is the modulus operator.

The next block in the architecture was a 2D-BLSTM^31,32^. The output from the final ResNet block was activated (with ELU) and normalized (using layer normalization) before being given as an input to the 2D-BLSTM. The number of nodes in each LSTM direction cell was ${D}_{BL}$. After the 2D-BLSTM, ${N}_{B}$ FC layers with ${D}_{FC}$ nodes were used, as per Block B in Fig. 1. The output of each FC layer was activated with the ELU function and normalized by using the layer normalization technique. A dropout rate of 50$\%$ was utilized for the hidden FC layers to avoid overtraining. The final stage of the architecture consisted of an output FC layer with one node and a sigmoidal activation function. The sigmoid function converts the output into the probability of each nucleotide being paired with other nucleotides. The number of outputs was equal to the number of elements in the upper triangular matrix of size $L\times L$, where $L$ is the length of the sequence.

Each model was implemented in Google’s Tensorflow framework (v1.12)^63^ and trained by using the ADAM optimization algorithm^64^ with default parameters. All models were trained on Nvidia GTX TITAN X graphics processing unit (GPU) to speed up training^65^. We trained multiple deep-learning models, based on the architecture shown in Fig. 1, on TR0 by performing a hyperparameter grid search over ${N}_{A}$, ${D}_{\rm{RES}}$, ${D}_{\rm{BL}}$, ${N}_{B}$, and ${D}_{\rm{FC}}$. ${N}_{A}$, ${D}_{\rm{RES}}$, ${D}_{\rm{BL}}$, ${N}_{B}$, ${D}_{\rm{FC}}$ were searched from 16 to 32, 32 to 72, 128 to 256, 0 to 4, and 256 to 512, respectively. These models were optimized on VL0 and tested on TS0. Transfer learning was then used to further train these models on TR1. During transfer learning, VL1 was used as the validation set and TS1 was used as an independent test set.

### Transfer learning

Transfer learning^35^ involves further training a large model that was trained on a large dataset for a specific task to some other related task with limited data. In this project, we used our large dataset bpRNA for initial training, and then transfer learning was employed by using the small PDB dataset as shown in Fig. 1. All of the weights/parameters that were learnt on TR0 were retrained for further training on TR1. During transfer learning, training and validation labels were formatted in exactly the same way as the initial training as a 2-dimensional (2D) $L\times L$ upper triangular matrix where *L* is the length of the RNA sequence. All of the labels used during the transfer learning were derived from high-resolution X-ray structures in the PDB. Some approaches in transfer learning freeze weights for specific layers and train for other layers. Here, we trained all the weights of the models without freezing any layer, as this provided better results. Previous work on protein molecular recognition features (MoRFs) prediction^36^ also showed that using transfer learning by retraining through all of the weights provides a better result than freezing some of the layers during retraining.

During transfer learning on TS1, we used the same hyperparameters (number of layers, depth of layers, kernel size, dilation factor, and learning rate) that were used for the TS0-trained models. All the models were validated for VL1, and based on the performance of these models on VL1, the 5 best models were selected for the ensemble. The parameters of these models are shown in Supplementary Table 9.

### Input

The input to SPOT-RNA is an RNA sequence represented by a binary one-hot vector of size *L* $\times$ 4, where *L* is the length of the RNA sequence and 4 corresponds to the number of base types (A, U, C, G). In one-hot encoding, a value of 1 was assigned to the corresponding base-type position in the vector and 0 elsewhere. A missing or invalid sequence in residue value of −1 was assigned in one-hot encoded vector.

This one-dimensional (*L* $\times$ 4) input feature is converted into two dimensional (*L* $\times$ *L* $\times$ 8) by the outer concatenation function as described in RaptorX-Contact^28^. The input is standardized to have zero mean and unit variance (according to the training data) before being fed into the model.

### Output

The output of our model is a 2-dimensional (2D) $L\times L$ upper triangular matrix where *L* is the length of the RNA sequence. This upper triangular matrix represents the likelihood of each nucleotide to be paired with any other nucleotide in a sequence. A single threshold value is used to decide whether a nucleotide is in pair with any other nucleotides. The value of the threshold was chosen in such a way that it optimizes the performance on the validation set.

### Performance measure

RNA secondary-structure prediction is a binary classification problem. We used sensitivity, precision, and F1 score for performance measure where sensitivity is the fraction of predicted base pairs in all native base pairs (${\rm{SN=TP/(TP+FN)}}$), precision is the fraction of correctly predicted base pairs (${\rm{PR=TP/(TP+FP)}}$), and F1 score is their harmonic mean (${\rm{F1=2(PR* SN)/(PR+SN)}}$). Here, TP, FN, and FP denote true positives, false negatives, and false positives, respectively. In addition to the above metrics that emphasize on positives, a balanced measure, Matthews correlation coefficient (MCC)^66^ was also used. MCC is calculated as

1 $${\mathrm{{MCC}}}=\frac{{\mathrm{{TP}}}\times {\mathrm{{TN}}}-{\mathrm{{FP}}}\times {\mathrm{{FN}}}}{\sqrt{({\mathrm{{TP}}}+{\mathrm{{FP}}})({\mathrm{{TP}}}+{\mathrm{{FN}}})({\mathrm{{TN}}}+{\mathrm{{FP}}})({\mathrm{{TN}}}+{\mathrm{{FN}}})}},$$

where TN denotes true negatives. MCC measures the correlation between the expected class and the obtained class. Moreover, a precision-recall (sensitivity) curve is used to compare our model with currently available RNA secondary-structure predictors. To show the statistical significance of improvement by SPOT-RNA over the second-best predictor, a paired *t* test was used on F1 score to obtain *P* value^67^. The smaller the *P* value is, the more significant the difference between the two predictors. As the output of the SPOT-RNA is a base-pair probability, we can use the ensemble defect as an additional performance metric. The ensemble defect describes the similarity between predicted base-pair probability and target structure^68^. It can be calculated by appending an extra column to the predicted probability matrix and target matrix for unpaired bases. If *P* and *S* are predicted and target structures, respectively, and *P*′ and *S*′ are predicted and target structures after appending the extra column, the ensemble defect (ED) is given by

2 $${\rm{ED}}=1-\frac{1}{L}\sum _{\begin{array}{c}i=1:L\\ j=1:L+1\end{array}}{P}_{ij}^{^{\prime} }{S}_{ij}^{^{\prime} },$$

where *L* is the length of the sequence. The smaller the value of ED is, the higher the structural similarity between predicted base-pair probability and target structure.

### Methods comparison

We compared SPOT-RNA with 12 best available predictors. We downloaded the stand-alone version of mxfold^33^ (available at https://github.com/keio-bioinformatics/mxfold), ContextFold^16^ (available at https://www.cs.bgu.ac.il/negevcb/contextfold/), CONTRAfold^14^ (available at http://contra.stanford.edu/contrafold/), Knotty^24^ (available at https://github.com/HosnaJabbari/Knotty), IPknot^23^ (available at http://rtips.dna.bio.keio.ac.jp/ipknot/), RNAfold^11^ (available at https://www.tbi.univie.ac.at/RNA/), ProbKnot^22^ (available at http://rna.urmc.rochester.edu/RNAstructure.html), CentroidFold^15^ (available at https://github.com/satoken/centroid-rna-package), RNAstructure^12^ (available at http://rna.urmc.rochester.edu/RNAstructure.html), RNAshapes^13^ (available at https://bibiserv.cebitec.uni-bielefeld.de/rnashapes), pkiss^13^ (available at https://bibiserv.cebitec.uni-bielefeld.de/pkiss), and CycleFold^27^ (available at http://rna.urmc.rochester.edu/RNAstructure.html). In most of the cases, we used default parameters for secondary-structure prediction except for pkiss. In pkiss, we used Strategy C that is slow but thorough in comparison with Strategies A and B that are fast but less accurate. For CONTRAfold and CentroidFold their performance metrics are derived from their predicted base-pair probabilities with threshold values from maximizing MCC.

### Reporting summary

Further information on research design is available in the Nature Research Reporting Summary linked to this article.

## Supplementary information

Supplementary Information

Reporting Summary

## Data Availability

The data used by SPOT-RNA for initial training (bpRNA)^34^ and transfer learning (PDB)^5^ along with their annotated secondary structure are publicly available at http://sparks-lab.org/jaswinder/server/SPOT-RNA/ and https://github.com/jaswindersingh2/SPOT-RNA.

## Code availability

SPOT-RNA predictor is available as a server at http://sparks-lab.org/jaswinder/server/SPOT-RNA/ and stand-alone software at https://github.com/jaswindersingh2/SPOT-RNA to run on a local computer. The web server provides an arc diagram and a 2D diagram of predicted RNA secondary structure through Visualization Applet for RNA (VARNA)^69^ tool along with a dot plot of SPOT-RNA-predicted base-pair probabilities.
