# Supplementary material for: RNA secondary structure prediction using an ensemble of two-dimensional deep neural networks and transfer learning
Source: Nat Commun. 2019 Nov 27;10:5407. doi: 10.1038/s41467-019-13395-9 (PMC6881452; doi:10.1038/s41467-019-13395-9)
Supplement: Supplementary file 1 — Supplementary Information [file 41467_2019_13395_MOESM1_ESM.pdf]

Supplementary Information for RNA Secondary Structure Prediction using an Ensemble of Two-dimensional Deep Neural Networks and Transfer Learning, by Singh et al.

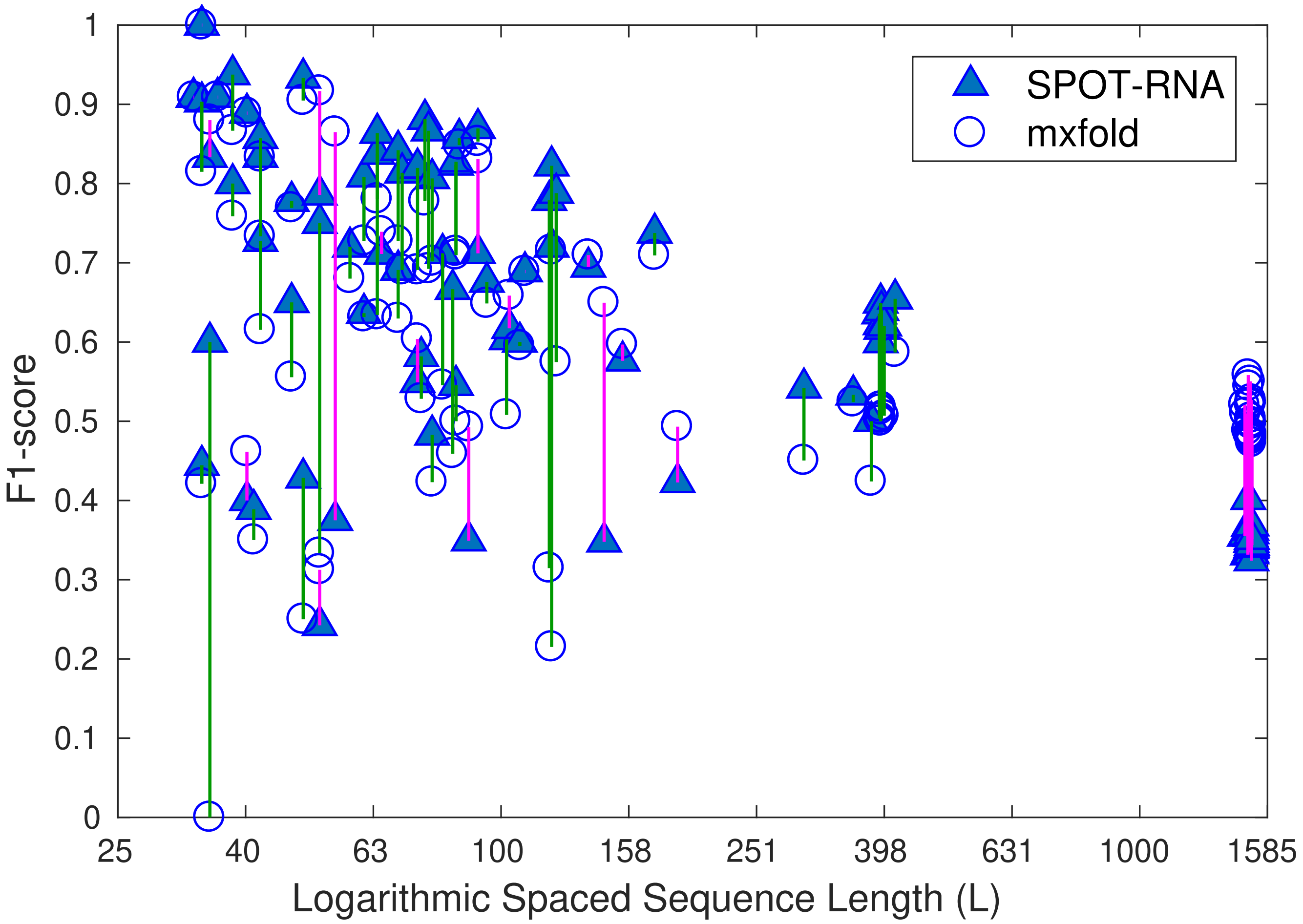

Supplementary Figure 1. SPOT-RNA improves over mxfold for the majority of RNAs except for those long RNAs (>1000). Comparison of SPOT-RNA and second best predictor (mxfold) for individual RNA in the test set of TS1 plus 32 long sequences. The color green indicates the improvement over mxfold by SPOT-RNA whereas the color magenta indicates the lack of improvement over to mxfold.

Supplementary Table 1. Performance of individual models and the ensemble on TS0 after initial training on TR0.

predictor	MCC[a]	F1[b]	Precision	Sensitivity	Accuracy
Model 0	0.617	0.618	0.642	0.596	0.998
Model 1	0.612	0.612	0.657	0.573	0.998
Model 2	0.593	0.591	0.654	0.537	0.998
Model 3	0.569	0.568	0.618	0.526	0.998
Model 4	0.611	0.605	0.705	0.531	0.995
Ensemble	0.629	0.626	0.709	0.550	0.999

[a] Matthews correlation coefficient. [b] Harmonic mean of precision and sensitivity.

Supplementary Table 2. Performance of 5-fold cross-validation on the combination of TR1+VL1 after transfer learning by individual models and their ensemble.

predictor	MCC[a]	F1[b]	Precision	Sensitivity	Accuracy
Model 0	0.671 (0.03[c])	0.659 (0.03[c])	0.830 (0.05[c])	0.547 (0.02[c])	0.995 (0.00[c])
Model 1	0.681 (0.02[c])	0.672 (0.03[c])	0.822 (0.02[c])	0.568 (0.03[c])	0.995 (0.00[c])
Model 2	0.679 (0.03[c])	0.673 (0.03[c])	0.798 (0.05[c])	0.583 (0.02[c])	0.995 (0.00[c])
Model 3	0.653 (0.03[c])	0.642 (0.03[c])	0.803 (0.06[c])	0.535 (0.02[c])	0.995 (0.00[c])
Model 4	0.667 (0.03[c])	0.657 (0.03[c])	0.811 (0.04[c])	0.553 (0.02[c])	0.995 (0.00[c])
Ensemble	0.701 (0.02[c])	0.690 (0.02[c])	0.853 (0.02[c])	0.580 (0.03[c])	0.995 (0.00[c])

[a] Matthews correlation coefficient. [b] Harmonic mean of precision and sensitivity. [c] Standard deviation based on five fold cross validation.

Supplementary Table 3. Performance on the independent test set TS1 after transfer learning by individual models and their ensemble during 5-fold cross-validation.

predictor	MCC[a]	F1[b]	Precision	Sensitivity	Accuracy
Model 0	0.662 (0.01[c])	0.647 (0.01[c])	0.831 (0.02[c])	0.530 (0.02[c])	0.994 (0.00[c])
Model 1	0.668 (0.01[c])	0.655 (0.01[c])	0.832 (0.03[c])	0.541 (0.01[c])	0.994 (0.00[c])
Model 2	0.666 (0.01[c])	0.656 (0.01[c])	0.810 (0.04[c])	0.554 (0.02[c])	0.994 (0.00[c])
Model 3	0.655 (0.00[c])	0.641 (0.00[c])	0.833 (0.02[c])	0.520 (0.01[c])	0.994 (0.00[c])
Model 4	0.647 (0.00[c])	0.636 (0.00[c])	0.809 (0.04[c])	0.531 (0.02[c])	0.994 (0.00[c])
Ensemble	0.690 (0.02[c])	0.687 (0.01[c])	0.888 (0.02[c])	0.562 (0.02[c])	0.995 (0.00[c])

[a] Matthews correlation coefficient. [b] Harmonic mean of precision and sensitivity. [c] Standard deviation based on five fold cross validation.

Supplementary Table 4. Performance comparison of all 5 models and their ensemble on TS1 with direct learning on TR1.

	Training Set	Analysis Set	MCC[a]	F1[b]	Precision	Sensitivity
Model 0	TR1	TS1	0.530	0.507	0.733	0.388
Model 1	TR1	TS1	0.500	0.497	0.601	0.424
Model 2	TR1	TS1	0.525	0.519	0.646	0.434
Model 3	TR1	TS1	0.533	0.500	0.782	0.367
Model 4	TR1	TS1	0.545	0.545	0.639	0.475
Ensemble	TR1	TS1	0.571	0.527	0.870	0.378

[a] Matthews correlation coefficient. [b] Harmonic mean of precision and sensitivity.

Supplementary Table 5. Performance comparison of all the predictors on the independent test set TS1 for non-canonical pairs, base triples, and lone pairs.

	F1 Non-Canonical	Precision Non-Canonical	Sensitivity Non-Canonical	F1 Triplets	Precision Triplets	Sensitivity Triplets	F1 Lone-Pair	Precision Lone-Pair	Sensitivity Lone-Pair
SPOT-RNA	0.255	0.732	0.154	0.085	0.118	0.067	0.138	0.638	0.078
mxfold	-	-	-	-	-	-	0.004	0.059	0.002
ContextFold	-	-	-	-	-	-	0.008	0.091	0.024
CONTRAfold	-	-	-	-	-	-	0.004	0.031	0.002
Knotty	-	-	-	-	-	-	0.008	0.182	0.004
IPknot	-	-	-	-	-	-	0.000	0.000	0.000
RNAfold	-	-	-	-	-	-	0.000	0.000	0.000
ProbKnot	-	-	-	-	-	-	0.000	0.000	0.000
CentroidFold	-	-	-	-	-	-	0.000	0.000	0.000
RNAstructure	-	-	-	-	-	-	0.000	0.000	0.000
RNAshapes	-	-	-	-	-	-	0.000	0.000	0.000
pkiss	-	-	-	-	-	-	0.000	0.000	0.000
CycleFold	0.173	0.184	0.163	-	-	-	0.046	0.025	0.025

Supplementary Table 6. Performance of all the predictors according to base-pair types on the test set TS2.

	All Base Pairs				Canonical Only			Watson-Crick Only			Wobble Only		
	MCC[a]	F1[b]	Precision	Sensitivity	F1[b]	Precision	Sensitivity	F1[b]	Precision	Sensitivity	F1[b]	Precision	Sensitivity
SPOTRNA	0.807	0.802	0.925	0.708	0.880	0.940	0.828	0.892	0.943	0.846	0.795	0.917	0.702
Knotty	0.789	0.783	0.913	0.686	0.876	0.913	0.841	0.902	0.926	0.879	0.675	0.797	0.585
RNAfold	0.788	0.781	0.919	0.679	0.874	0.919	0.833	0.891	0.923	0.862	0.741	0.882	0.638
RNAshapes	0.787	0.780	0.926	0.674	0.873	0.926	0.826	0.895	0.934	0.858	0.708	0.851	0.606
RNAstructure	0.778	0.772	0.910	0.670	0.864	0.910	0.822	0.880	0.914	0.848	0.744	0.871	0.649
CONTRAfold	0.776	0.771	0.896	0.677	0.862	0.896	0.830	0.881	0.902	0.860	0.720	0.843	0.628
pkiss	0.774	0.769	0.892	0.676	0.859	0.892	0.829	0.881	0.902	0.860	0.699	0.806	0.617
ProbKnot	0.765	0.760	0.889	0.664	0.850	0.889	0.814	0.867	0.897	0.838	0.726	0.824	0.649
mxfold	0.763	0.753	0.915	0.640	0.845	0.915	0.786	0.872	0.931	0.820	0.646	0.776	0.553
IPknot	0.763	0.752	0.924	0.634	0.845	0.924	0.777	0.863	0.927	0.807	0.701	0.900	0.574
ContextFold	0.750	0.739	0.910	0.623	0.830	0.910	0.764	0.859	0.922	0.804	0.605	0.793	0.489
CentroidFold	0.746	0.732	0.927	0.605	0.824	0.927	0.742	0.846	0.929	0.776	0.653	0.906	0.511
CycleFold	0.686	0.690	0.704	0.677	0.764	0.779	0.750	0.783	0.783	0.784	0.613	0.742	0.521

[a] Matthews correlation coefficient. [b] Harmonic mean of precision and sensitivity.

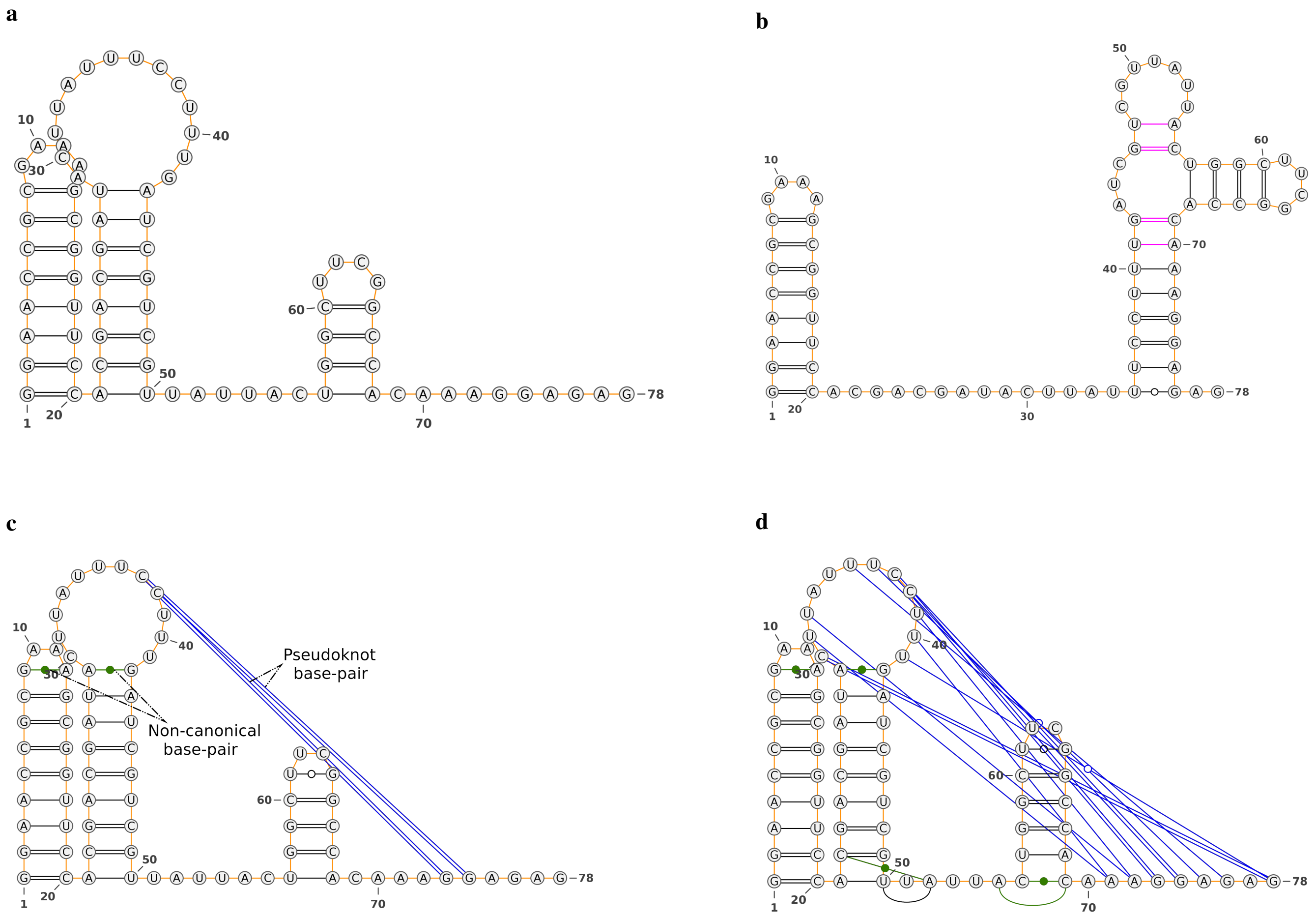

Supplementary Figure 2. Comparison of structure predicted by mxfold, IPknot, and SPOT-RNA with the native structure of a Riboswitch RNA. The secondary structure of a Riboswitch RNA (chain A in PDB ID 4jf2) represented by 2D diagram with canonical base-pair (BP) in black color, non-canonical BP in green color, pseduoknot BP and lone-pair in blue color, and wrongly predicted BP in magenta color: **a** by mxfold, with 100% precision and 54% sensitivity, **b** by IPknot with 82% precision and 49% sensitivity, **c** predicted by SPOT-RNA with 100% precision and 68% sensitivity, **d** native structure.

Supplementary Table 7. The number of different types of base-pairs in the datasets.

	No. of RNAs	Canonical Base-Pairs		Non-canonical Base-Pairs	Pseudoknot Base-Pairs	Multiplets Base-Pairs	Average sequence length	Maximum sequence length
		Watson-Crick	Wobble					
TR0	10,814	260,601	37,194	34,327	9,644	-	133	500
VL0	1,300	30,873	4,428	4,095	1,001	-	131	500
TS0	1,305	32,102	4,599	4,082	1,206	-	136	500
TR1	120	2,934	316	911	312	1,194	78	414
VL1	30	694	84	226	71	301	77	159
TS1	67	1,554	180	493	160	406	75	189
TS2	39	643	94	167	21	230	51	155

Supplementary Table 8. The numbers of base pair in the stem, pseudoknots and number of nucleotides in different secondary structure types.

	No. of RNAs	No. of RNAs with at least 1 PK	Pseudoknot Base-Pairs	No. of Base Pairs in Stem	No. of Nucleotides in Hairpin Loop	No. of Nucleotides in Bulge	No. of Nucleotides in Multiloop	No. of Nucleotides in Internal Loop
TR1	120	80	312	2529	1341	381	853	443
VL1	30	22	71	652	288	83	125	208
TS1	62	40	144	1470	582	211	263	389

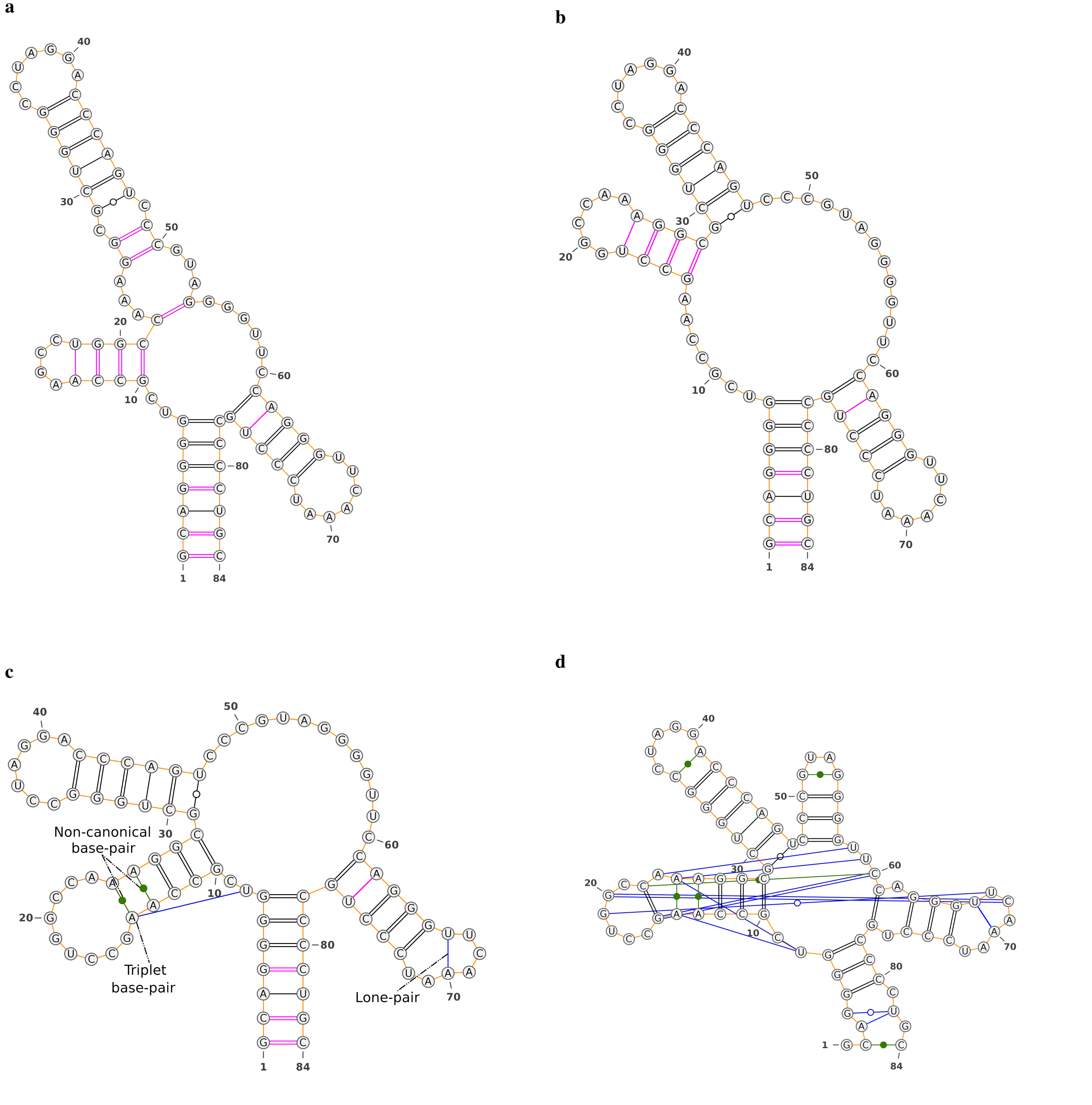

Supplementary Figure 3. Comparison of structure predicted by mxfold, IPknot, and SPOT-RNA with the native structure of a transfer RNA. The secondary structure of a transfer RNA (chain B in PDB ID 2zzm) represented by 2D diagram with canonical base-pair (BP) in black color, non-canonical BP in green color, pseduoknot BP and lone-pair in blue color, and wrongly predicted BP in magenta color: **a** by mxfold, with 56% precision and 39% sensitivity, **b** by IPknot with 63% precision and 39% sensitivity, **c** predicted by SPOT-RNA with 84% precision and 58% sensitivity, **d** native structure.

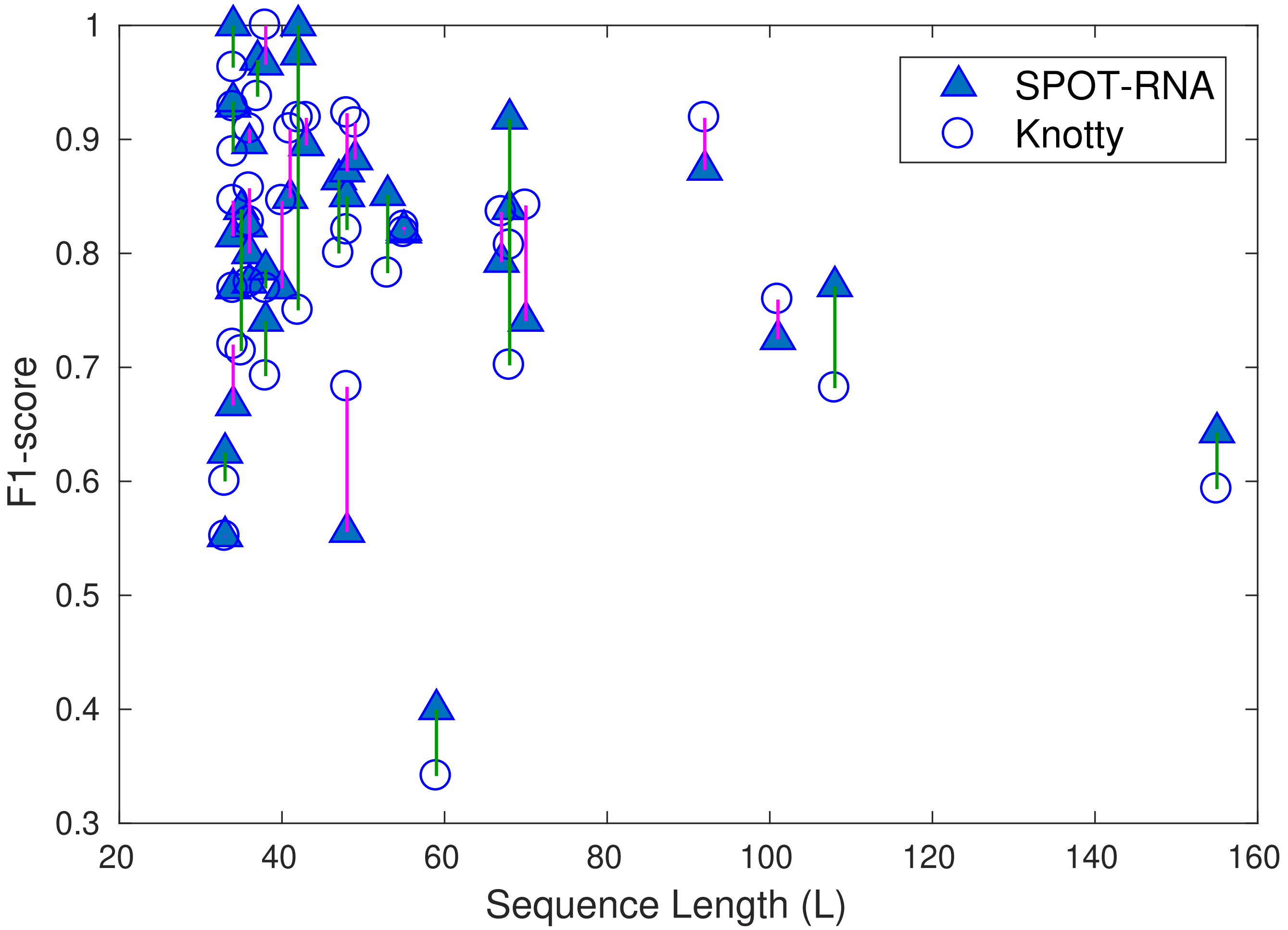

Supplementary Figure 4. Comparison of SPOT-RNA and second best predictor (Knotty) for individual RNA in the test set of TS2. The color green indicates the improvement over Knotty by SPOT-RNA whereas the color magenta indicates the lack of improvement over Knotty. For this easy dataset of 39 RNAs, SPOT-RNA is only marginally better than Knotty but statistically significant better over other methods.

Supplementary Table 9. SPOT-RNA ensemble architectures.

	Num. blocks/layers			Depth of Layer			Dilation Factor
	N_A	N_{BL}	N_B	D_{RES}	D_{BL}	D_{FC}	
Model 0	16	-	2	48	-	512	-
Model 1	20	-	1	64	-	512	-
Model 2	30	-	1	64	-	512	-
Model 3	30	1	-	64	200	-	-
Model 4	30	-	1	64	-	512	$2^{i\%5}$
