# Supplementary material for: RNA secondary structure prediction using an ensemble of two-dimensional deep neural networks and transfer learning
Source: Nat Commun. 2019 Nov 27;10:5407. doi: 10.1038/s41467-019-13395-9 (PMC6881452; doi:10.1038/s41467-019-13395-9)
Supplement: Supplementary file 2 — Reporting Summary [file 41467_2019_13395_MOESM2_ESM.pdf]

nature research

Corresponding author(s): Yaoqi Zhou

Last updated by author(s): Oct 14, 2019

Reporting Summary

Nature Research wishes to improve the reproducibility of the work that we publish. This form provides structure for consistency and transparency in reporting. For further information on Nature Research policies, see Authors & Referees and the Editorial Policy Checklist.

Statistics

For all statistical analyses, confirm that the following items are present in the figure legend, table legend, main text, or Methods section.

n/a	Confirmed	
☒	☐	The exact sample size (*n*) for each experimental group/condition, given as a discrete number and unit of measurement
☒	☐	A statement on whether measurements were taken from distinct samples or whether the same sample was measured repeatedly
☒	☐	The statistical test(s) used AND whether they are one- or two-sided<br>*Only common tests should be described solely by name; describe more complex techniques in the Methods section.*
☒	☐	A description of all covariates tested
☒	☐	A description of any assumptions or corrections, such as tests of normality and adjustment for multiple comparisons
☐	☒	A full description of the statistical parameters including central tendency (e.g. means) or other basic estimates (e.g. regression coefficient) AND variation (e.g. standard deviation) or associated estimates of uncertainty (e.g. confidence intervals)
☐	☒	For null hypothesis testing, the test statistic (e.g. *F*, *t*, *r*) with confidence intervals, effect sizes, degrees of freedom and *P* value noted<br>*Give P values as exact values whenever suitable.*
☒	☐	For Bayesian analysis, information on the choice of priors and Markov chain Monte Carlo settings
☒	☐	For hierarchical and complex designs, identification of the appropriate level for tests and full reporting of outcomes
☒	☐	Estimates of effect sizes (e.g. Cohen's *d*, Pearson's *r*), indicating how they were calculated

Our web collection on statistics for biologists contains articles on many of the points above.

Software and code

Policy information about availability of computer code

Data collection	Datasets were directly downloaded from proteins data bank and bpRNA webserver. To remove sequence identity infernal 1.1.2 with 80% sequence identity cutoff was used. To further remove sequence identity Blastn 2.6.0+ was used.
Data analysis	To obtain RNA secondary structure motifs/types, the bpRNA program (available at https://github.com/hendrixlab/bpRNA) was used.

For manuscripts utilizing custom algorithms or software that are central to the research but not yet described in published literature, software must be made available to editors/reviewers. We strongly encourage code deposition in a community repository (e.g. GitHub). See the Nature Research guidelines for submitting code & software for further information.

Data

Policy information about availability of data

All manuscripts must include a data availability statement. This statement should provide the following information, where applicable:
- Accession codes, unique identifiers, or web links for publicly available datasets
- A list of figures that have associated raw data
- A description of any restrictions on data availability

The data used by SPOT-RNA for training, validation and testing is publicly available at 'http://sparks-lab.org/jaswinder/server/SPOT-RNA/' and github (https://github.com/jaswindersingh2/SPOT-RNA).

Field-specific reporting

Please select the one below that is the best fit for your research. If you are not sure, read the appropriate sections before making your selection.

☒ Life sciences ☐ Behavioural & social sciences ☐ Ecological, evolutionary & environmental sciences

For a reference copy of the document with all sections, see nature.com/documents/nr-reporting-summary-flat.pdf

Life sciences study design

All studies must disclose on these points even when the disclosure is negative.

Sample size	The sample size of training, validation, and test set were determined by the number of non-redundant RNAs in Protein Data Bank and the bpRNA web server according to CD-HIT-EST and BLAST-N tool. We employed all the non-redundant randomly distributed RNAs for training, validation, and testing.
Data exclusions	All the sequences in test sets homologous to training data (according to CD-HIT-EST and BLASTN tools) were removed to avoid any kind of potential bias during testing.
Replication	All the training weights/parameters of the models were saved during experiments. Now, these trained parameters are deployed at web server for results replication. All these model parameters are available as standalone software to run on a local computer for results replication.
Randomization	All the training sequences were randomly split into training, validation, and test data sets.
Blinding	The test sets used for SPOT-RNA model performance measures were completely independent of data used for model training. CD-HIT-EST and BLAST-N tools were used to separate test sets from training data.

Reporting for specific materials, systems and methods

We require information from authors about some types of materials, experimental systems and methods used in many studies. Here, indicate whether each material, system or method listed is relevant to your study. If you are not sure if a list item applies to your research, read the appropriate section before selecting a response.

Materials & experimental systems

n/a	Involved in the study
☒	☐ Antibodies
☒	☐ Eukaryotic cell lines
☒	☐ Palaeontology
☒	☐ Animals and other organisms
☒	☐ Human research participants
☒	☐ Clinical data

Methods

n/a	Involved in the study
☒	☐ ChIP-seq
☒	☐ Flow cytometry
☒	☐ MRI-based neuroimaging
